# Prediction of Orthodontic Extraction Decisions Using Machine Learning Algorithms: A Retrospective Study

**DOI:** 10.4317/jced.64288

**Published:** 2026-07-29

**Authors:** Alah Dawood Aldawoody, Shehab Ahmed Hamad

**Affiliations:** 1 Assistant professor of Orthodontics, Department of Pedodontics, Orthodontics and Preventive Dentistry, College of Dentistry, University of Mosul, Mosul, Iraq; 2 Professor of Oral and Maxillofacial Surgery, Kurdistan Higher Council of Medical Specialties, Erbil, Iraq

## Abstract

**Background:**

Orthodontic extraction decision-making remains difficult and highly subjective, particularly in marginal cases where the clinicalcues are ambiguous. Objectives: To design machine learning (ML) models for prediction of extraction vs. non-extraction decision-making and estimate the influenceof key clinical predictors on such decisions.

**Material and Methods:**

Retrospective analysis was performed on 120 patients with extraction and 80 patients without extraction from asample of pretreatment records over 2 years. Five ML models including Logistic Regression (LR), Random Forest (RF), Support VectorMachine (SVM), Decision Tree (DT) and XGBoost are employed in this research by applying Python's Scikit-learn. The datasetwas divided in two parts for training and testing at a ratio of 70:30. The sensitivity, specificity, accuracy and AUC-ROCwere used to evaluate and compare the performance of the models. In order to rank the most important features for decision-making, feature importance was calculated.

**Results:**

RF model provided the highest accuracy (93.5%) and AUC-ROC (0.95) values, whereas XGBoost was the second-bestmodel, with accuracy (90.2%) and AUC-ROC (0.92). Mandibular crowding (weight = 0.28) and IMPA (L1-MP angle,weight = 0.22) were the most influential predictors.

**Conclusions:**

Ensemble ML models, in particular RF, yield a promising objective methodology for clinical decision support in orthodontics topotentially lessen inter-clinician variation and enhance consistency in treatment planning.

## Introduction

The selection for or against extraction of permanent teeth in orthodontics remains one of the most critical and most contentiously debated issues in treatment planning (1 , 2). Such a selection affects the patient's profile, periodontal health, retention of teeth and satisfaction in the long term (3 , 4). Orthodontic practice has oscillated from one extreme ideology of non-extraction expansionist philosophy where Edward Angle advocated for retention of all permanent teeth and the extraction therapy ideologies (Tweed, Begg) which recognized limitations to dento-alveolar expansion (5 , 6). This verbal debate has resulted in the modern day practitioner trying to decode an intricate diagnostic problem with minimal clear-cut indicators (7). In so-called "borderline" cases, in which crowding is mild to moderate, facial appearance is somewhat inconclusive, and cephalometric measurements lie between two demarcatednumbers, low inter-clinician agreement is a common occurrence (8). In the borderline cases, even experienced orthodontists are unsure as to whether to treat a case orthodontically with extractions or without extractions in approximately 20-25% of patients (9 , 10). These variations can cause disparities in the management of patients and raise the question regarding how objective traditional treatment planning really is (11). Injudiciously selected extractions may also lead to irreversible damages such as the aesthetic compromise of a dentofacial appearance (e.g. Marked lip retrusion, flattened profile) iatrogenic damage to the periodontium (e.g. Root exposure, fenestration) and long term instability (12 , 13). Artificial intelligence (AI), and morerecently machine learning (ML), provide a possible answer to the clinical puzzle (14). ML-based techniques are unlike classical statistics which need to be based on previous assumptions about linear or non-linear relationships; ML is able to model non-linear, multivariate relationships between high-dimensional data (15 , 16). By learning from previous choices made by an expert, ML can be a more objective second opinion, which may help remove human subjectivity from clinical reasoning (17 , 18). Over the last few years, many papers have explored the prediction of orthodontic extractions using ML with good results (4 , 19). For instance, Suhail et al. Obtained an accuracy of 91% by designing ensemble learning classifiers (Random Forest and XGBoost) with data from cephalometric and dental casts. (20), whereas Mason et al. used deep learning on lateral cephalograms with an AUC of 0.85 (21), and Jung and Kim reached an accuracy of approximately 89% using neural networks with 12 variables (5). Recently Huang et al. attained an accuracy of 95.97% by employing an XGBoost model, demonstrating that ensemble methods outperform individual models in comparative analyses, and pinpointed overjet, overbite, and mandibular crowding as the key contributing factors (10), and Real et al. have shown the possibility of automated extraction prediction with an AI-based approach, showing an accuracy of 95.2% with a sensitivity of 97.2% and a specificity of 93.1%. (22). Nevertheless, additional research remains necessary since some earlier studies have been constrained by small sample sizes or designs centered on a single location (5 , 23), whereas other investigations have concentrated on particular treatment methods like clear aligner therapy (24). Little evidence exist that there is a clear hierarchy among the clinical features responsible for the decision of extraction in an ML-based derivation (25 , 26). Knowing the hierarchy is important for transparency, education and validation of ML models (27). Lacking this interpretability, clinicians might continue to be skepticalof "black box" AI recommendations (28). The current study is the first that attempts to: (1) develop and test five different ML designs (Logistic Regression, Random Forest, Support Vector Machine, Decision Tree, and XGBoost) based on a robust sample of n= 200 of orthodontic records prior to treatment, (2) evaluate their predictive performance using a variety of performance measures (accuracy, sensitivity, specificity, AUC-ROC), and (3) identify the clinical factors having the greatest impact on automated extraction decision making to increase interpretability and clinical acceptance of AI-based treatment planning.

## Material and Methods

- Study Design and EthicalConsiderations This single-center retrospectivestudy was approved by the Institutional Review Board. Asit is a retrospective study, patient consent was waived by the IRB and all patient data were blinded before analysis. - Sample Size Estimation andPower Analysis Prior to the study, sample size calculations wasdone using G*Power software (version 3.1.9.7, Heinrich Heine University, Düsseldorf, Germany). To achievesignificant agreement between the ML model and the human expert gold standard (anticipated Cohen's Kappa > 0.70) at an level of 0.05 and a statistical power of 0.80, a sample size of at least 184 cases was necessary. Therefore, 200 pretreatment recordswere recruited in order to compensate for the missing data or exclusion. - Selection criteria Inclusion criteria were: (1) a full setof pretreatment records that included lateral cephalometric radiograph, dental casts or digital models, extraoral facial photographs; (2) permanent dentition (excluding third molars); (3) no history of previous orthodontic therapy; (4) no craniofacial abnormalities (e. g. , cleft lip/palate, craniosynostosis); and (5) treatment was performed by a solitary seasoned orthodontist (>15 years of clinical experience) to keep the gold standard decision consistent. Exclusion criteria included: (1)any missing data of the 25 predictor variables; (2) tooth extraction history for caries or trauma; (3) syndrome or cleft patients; and (4) cases with orthognathic surgery. - Data Collection and Variable Definition Two trained reviewers extracted thedata from the electronic records (intraclass correlation coefficient > 0.90 among all continuous variables). A totalof 25 variables were registered and grouped as follows: Cephalometric variables (n=8): ANB, SNB, FMA, IMPA(L1-MP angle), U1-NA (mm and °), the interincisal angle, and the mandibular plane to SN angle. Clinical and model-derived variables (n=12): mandibular crowding (mm, derived from subtraction on dentalcasts), maxillary crowding (mm), overjet (mm), overbite (mm), arch form (square, tapered, ovoid), molar relationship (Angle Class I, II, III), depth of the curve of Spee (mm), tooth-size disparity (Bolton disparity, mm), midline deviation (mm), amount of erupted teeth without third molars, presence of impacted teeth, and arch perimeter disparity (mm). Soft-tissue and demographic variables (n=5): Nasolabialangle, lip competence (competent, incompetent with mentalis strain), facial profile (convex, straight, concave), age (years), and sex (male/female). Each continuous variable wasindependently measured twice by 2 researchers, and the average was used for analysis. Discussion served to achieve consensus regarding categorical variables or a third reviewer. - Outcome variable (Gold standard) The dependent variable was extraction (at least one permanent tooth extracted for orthodontic purposes other thanthird molars) or non-extraction. An expert orthodontist (over 15 yearsof experience) who performed the pretreatment analysis was responsible for the gold standard decision, which was based on an assessment of cephalometrics, study models, facial photographs, and clinical judgment. This person didnot know the predictions of the ML models. - Data Preprocessing Missing data were present in <5% of all entries and were handled using k-Nearest Neighbors (k-NN) imputation with k=5, as this method has been shown to preserve data distribution better than mean imputation in orthodontic datasets. After imputation, all continuous variables were standardized (z-score normalization) to ensure that variables with larger magnitudes did not dominate model training. This transformation subtracts the mean and divides by the standard deviation for each variable. Categorical variables (e.g., arch form, molar relationship, lip competence) were one-hot encoded. - Data Splitting and Cross-Validation The whole sample (N=200) was divided into a training set (70%, n=140) and a test set (30%, n=60) via stratified random sampling so that the ratio of extraction/non-extraction (roughly 60:40) wasmaintained in both sets. Hyperparameter tuning was performed with a five-fold cross-validationwithin the training set. In this method, the training set is divided into five equal portions ("folds"); the model is trainedwith four folds and tested on one fold, rotating through each fold as the test set. The final performance of the modelwas then assessed on the unseen test set. - Machine Learning Algorithms Five algorithms were implemented using Python (version 3.10.12) and the Scikit-learn library (version 1.2.2): 1. Logistic Regression (LR): A linear probabilisticclassifier with logit as the link function. We usedL2 regularization (Ridge) with the default hyperparameter C=1.0 after grid search (C values: 0.01, 0.1, 1, 10). 2. Random Forest (RF): An ensemble of decision trees usingbagging. Tunedhyperparameters: number of trees (n_estimators = 100, 200, 300), maximadepth (5, 10, 15, none), and minimum samples split (2, 5, 10). 3.Support Vector Machine (SVM): Akernel based classifier with RBF kernel. Hyperparameters tuned: C(0.1, 1, 10, 100) and gamma (scale, auto, 0.01, 0.1). 4. Decision Tree (DT): Anindividual tree classifier with Gini impurity as the splitting criterion. Tuned hyperparameters:max_depth = [3,5,10,none] and min_samples_leaf = (1 , 2 , 4). 5. XGBoost: A gradient-boosted ensemble algorithm that is considered state of the art forgeneralized linear models when dealing with sparse data and regularization. Implemented via the xgboostmodule (version 1.7.5). Hyperparameters tuned: learning_rate (0.01, 0.1, 0.2), n_estimators (100, 200, 300), max_depth (3,5, 7). Thehyperparameters were tuned via grid search with fivefold cross-validation on the training set (16). Thebest hyperparameter setting was chosen according to the validation accuracy. The models were then retrainedon the full training set with the best parameters and tested on the hold-out test set. - Model Evaluation Metrics The performance of the models was evaluated using the following parameters based on confusion matrix: (28) 1. Accuracy= (TP+TN) / (TP + TN + FP + FN) 2. Sensitivity (recall rate): TP / (TP + FN) - the extractor's ability to extract cases correctly 3. Specificity: TN / (TN + FP) - the extractor's ability to extract cases correctly 4. AUC-ROC: the area under the receiver operating characteristic curve, a plot of true positive rate versus false positive rate at various threshold levels. AUC of 1.0 means perfect discrimination and 0.5 meansrandom chance. Feature importance in tree- based models (RF, XGB, DT) were calculated as the mean decrease in gini impurity. For LR, coefficients were standardizedto represent relative importance. Statistical analysiswas done in Python 3.10.12 and significance of difference in performance between models was evaluated using McNemar's test with p < 0.05 indicating significance.

## Results

**Figure 1:**
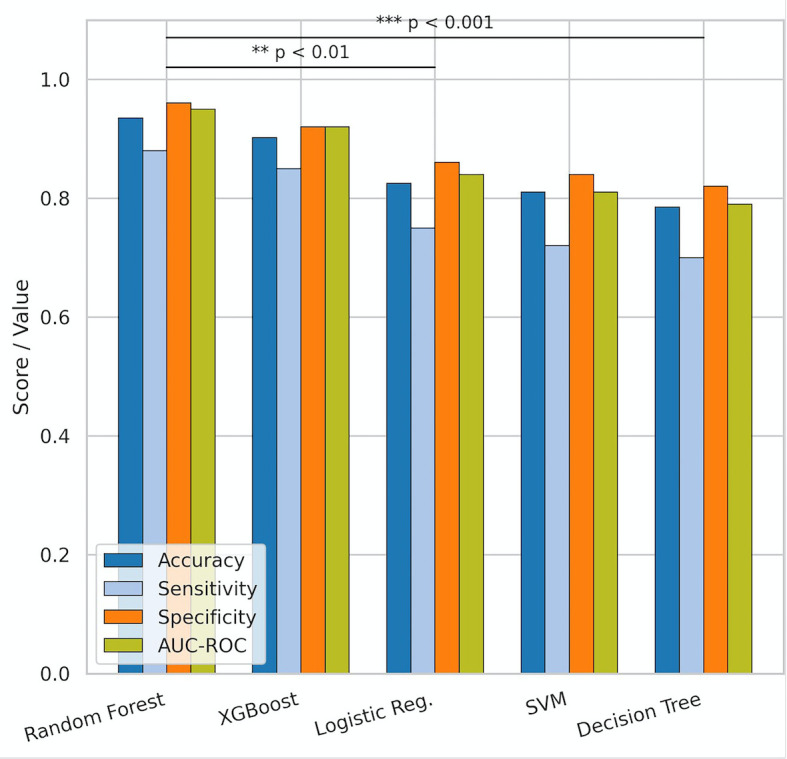
Model performance comparison.

The accuracy, sensitivity,specificity and AUC-ROC were used to evaluate the performance of the models. The ensemble models also showed a clear superiorityin diagnostic accuracy compared to the single classifiers. Random Forest outperformed with an accuracy of 93.5 % andspecificity of 0.96, suggesting high performances in true prediction of 'non-extraction'. Itssensitiviry (0.88) was also high, which means the method could well detect the extraction cases. XGBoost was a close second in performance, with an accuracy of90.2% and an AUC-ROC of 0.92. Linear models (LR, SVM) and thesingle DT also proved to be credible classifiers, but were not competitive with the ensemble approaches. The accuracy difference between Random Forest and XGBoost was not statistically significant according to McNemar's test (p= 0.12), but both were significantly better than LR (p < 0.01), SVM (p < 0.01) and DT (p < 0.001), as shown in Figure 1.

1

**Figure 2:**
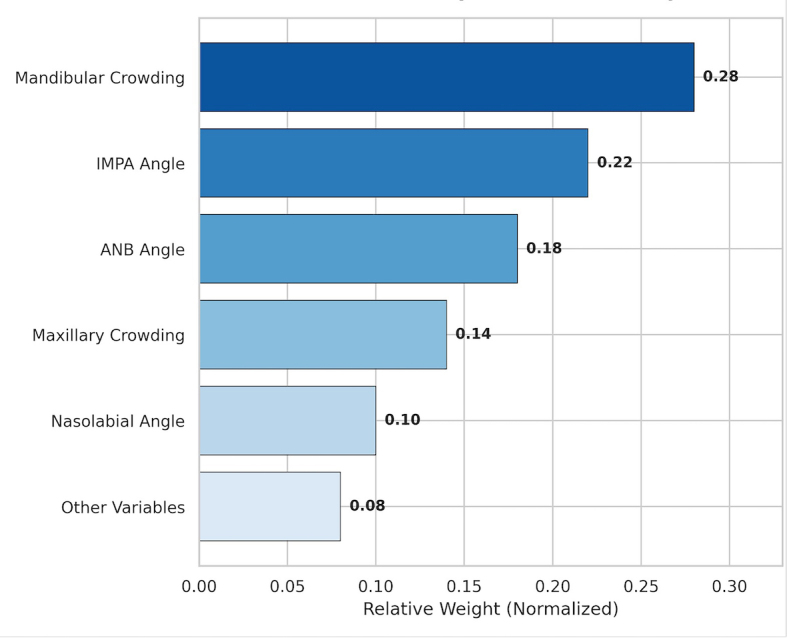
Feature importance hierarchy.

Mandibular crowding was the most influential variable in the extraction decision, with a relative weight of 0.28. The IMPA angle (lower incisor inclination) followed closely at 0.22, highlighting the importance of incisor proclination. The ANB angle (sagittal skeletal relationship) contributed 0.18, while maxillary crowding had a weight of 0.14. Nasolabial angle (soft tissue profile) and other variables carried lower weights at 0.10 and 0.08, respectively, indicating they played a comparatively smaller role in the decision-making process. The relative weights(normalized to sum to 1.0) are included in Figure 2.

2

## Discussion

The findings of this study demonstrate that ensemble ML algorithms, especially Random Forest, can closely mimic the complex multidimensional diagnosticthinking of experienced orthodontists with very good agreement. An accuracy of 93.5% shows that the DL has been able to learn the weighted relationships of the clinical features thatgovern whether a case is non-extraction or extraction (space closure) through tooth removal. Such a level of performance is encouraging not only fromthe technical point of view but also from the clinical perspective, since it could have the imply that ML-based decision support systems may in the future become as reliable "second opinions" in routine orthodontics practice, most especially for borderline cases in which human experts often have diverging opinions (22). Notably, both ensemble methods (RF and XGBoost) outperformed substantially the linear models (Logistic Regression andSVM) and single DT. This is consistent with the studies of Köktürk et al. (2024) and Suhail et al. (2020), which suggestedthat ensemble-based methods can more effectively capture non-linear interactions and higher-order relations between features (19 , 20). For instance a moderate mandibular crowding and a slightly increased IMPAmay be relatively innocuous on its own, yet an exponential increase on extrusion probability when the two are combined (10 , 23). One of the significant strengths of RFis that it can naturally model such interactions, without obviously defined changes in the covariates. The feature importance hierarchy also provides some interesting clinical considerations that are in line with what is already known in orthodontics as well as with some subtle additions that are not immediately obvious to the newer clinicians. The single biggest contributor was crowding of the mandibular arch ( weight of 0.28) (20 , 27). Our result then is powerful evidence for the monkey-in-the-hand clinical primitives that the lower arch is the single most restrictive determinant in orthodontic treatment planning. Anatomically, the symphysis of the mandible along with its buccal and lingual cortical plates, are much harder to expand compared to the maxilla which can be expanded through the midpalatal suture. Hence, cases with significant mandibular crowding (typically > 4-5 mm of space deficit), non-extraction solutions like expansion, IPM reduction, or distalization of the molars are either mechanically impossible or biologically risky (14). To try and correct crowding by proclining lower incisors beyond the alveolar housing, can result in iatrogenic complications such as fenestrations, gingival recession, dehiscence, and even root resorption (12 , 21). The model correctly learnt this biological limitation and suggested extraction. The second most important predictor was IMPA (Incisor Mandibular Plane Angle), with a weight of 0.22. This variable correspondsdirectly to the amount of lower incisor proclination or retroclination in relation to the mandibular plane (23). Greater IMPA (>95-100°) means that the lower incisors is already proclined, possibly owing tocompensation for skeletal discrepancy (11). Moreover, insuch patients, it would be hazardous to recommend additional proclination to alleviate crowding as it would push the incisors outside the alveolar bone limits, impair lip competence, and may aggravate gingival recession. On the other hand, a low IMPA (<85°) points to thepossibility of retroclined incisors, which in some cases can be safely proclined to gain space, without extractions (19). The model's over-emphasis on IMPA suggests that it learned these biological limits of dental compensation, paralleling the thought process of experienced clinicians who assess incisor inclination when considering an extraction or non-extraction approach. The third ranked parameter in importance was the ANB angle which had a weight of 0.18, emphasizing the importance of the sagittalskeletal relationship. High ANB (<5°) in Class IIpatients may represent maxillary protrusion or mandibular retrusion (20). Extraction of premolars (mostly maxillary first premolars) may be essential in such situations to facilitate retraction of the anterior segment to enhance theprofile and to attain a functional overjet (11). Negative ANB (<0°) in Class III patients may similarly necessitate extraction of lower premolars for dental camouflage, though surgical treatment may alsobe undertaken (27). The fact that this model included ANB as a one of the top 3 variables shows skeletal pattern is still one of the most essential diagnosis components for orthodontics even on an AI model (23). Paradoxically the maxillary crowding was given a slightly lower coefficient weight than mandibular crowding (0.14). This has a clinical rationale as the maxilla is a bit more treatable for expansion by the midpalatal suture and that also is more treatment effective in growing patients than in adults. Consequently, the maxillary crowding is treated more frequently with a non-extraction than mandibular crowding (19). Thenasolabial angle (weight, 0.10) was still among the top five predictors. This is a very refined portion of the model's learning in that it actually embodies the idea of "face-driven" orthodontic treatment planning-an ideal that is taught but difficult toquantify. The nasolabialangle is the soft-tissue analogue for the sagittal position of the maxillary incisors and lip support (12). A reduced nasolabial angle is often associated with forwardly positioned incisors and a convex profile, where extraction and incisor retraction may enhance esthetics by enlarging the angle. Conversely, an increased nasolabial angle tends to be associated with a retruded profile and thin lips (12). In thesecases extractions are contraindicated since further retraction would overflatten the face, signaling toward an aged or "dished-in" appearance (12 , 27). The fact that the model was able to learn this soft-tissue boundary without being explicitly programmed to do so demonstrates the value of the training data and the power of ensemble learning. In comparing our findings to the current literature, various aspects merits attention. Hatoum et al. (2026) found 90% accuracy using a neural network on 300 images; however, their model depended solely on intraoral photos and excluded cephalometric variables or dental cast crowding measurements, potentially restricting its generalizability to intricate orthodontic scenarios (25). Jung and Kim (2016) found an accuracy rate of around 89% utilizing a neural network with 12 variables, but the limited sample size (n=94) could suggest potential issues with overfitting (5). Mason et al. (2023) applied a Random Forest model to lateral cephalogram data from 1,365 patients, obtaining an AUC of 0.85, which is below our XGBoost (0.92) and Random Forest (0.95) models (21). Our method advantages from using structured clinical variables that clinicians can easily interpret, in contrast to deep learning methods that function on unprocessed images and are commonly seen as a 'black box' (17 , 26). Real et al. (2022) assessed various predictive models and discovered that integrating dental cast and radiographic information resulted in 95.2% accuracy (sensitivity: 97.2%, specificity: 93.1%), whereas using only cephalometric data produced a mere 72.7%, showcasing the essential importance of including dental cast measurements (22). The clinical impact of this investigation is substantial. the ranking in our study presents a straightforward, evidence-based ranking to teach orthodontic residents training to use it or to facilitate clinical reasoning. Clinicians with limited experience may have difficulty determining which of the numerous competing pieces of diagnostic information to prioritize; understanding that mandibular crowding and IMPA jointly account for 50% of the predictive weight might help them concentrate first on these two important variables before moving on to tertiary aspects of the case (10 , 19). Second, the good performance of ensemble algorithms also imply that ML-based CDSS may be successfully applied for clinical use as a trustworthy second opinion. In borderline cases, inwhich the treating orthodontist might feel uncertain, the CDSS could give a recommendation based on rules that it had learned from previous expert decisions. This in turn might decrease inter-clinician variability, increase standardization, and even decrease the rates of iatrogenic errors. Third, because the model is completely transparent with respect to feature importance, clinicians canalways audit its advice. Should the AI recommend extraction, it provides an opportunity for the clinician to check if mandibular crowding and IMPA are indeed high. If they aren't then it would be appropriate for the clinician to disagree with the AI recommendation and disregard it. This is considered a clinician supervised solution where the clinician maintains a level of authority and the strengths of AI, which include pattern recognition, are utilized. Nevertheless, this study has several limitations that must be acknowledged. The retrospective nature may cause selection bias, since the sample consists of the cases treated by one single orthodontist at one single institution. Although this provided a high butconsistent gold standard, this may be not capture all variability in treatment philosophies of other treating practitioners or in other countries or within different healthcare systems. Although regarded as the gold standard, expert clinical decisions are not inherently synonymous with optimal biological or long-term outcomes. Lacking longitudinal data on the stability of the post-treatment, periodontal health, root resorption and patient satisfaction, we cannot say that the model's guidelines yield better outcomes than any other strategies. Though the n=200 case dataset is sufficiently powered for the primaryanalysis, it is small for more elaborate deep learning architectures that might draw additional benefits from thousands of examples. We also did not use three dimensional imaging (e.g., CBCT) which may allow a more precise evaluation of alveolar bone volume, cortical plate thickness androot proximity which could impact extraction decisions in severe cases. The analysis did not consider patient preference, or cost of treatment or length of the treatment itself, allreal world determinants that affect the decision to extract teeth in clinical practice. Future studies should overcome these limitations with multiple directions. Our findings should be further validated by prospective multicenter studies with a large number of samples to investigate whether they can be generalized todifferent populations, treatment philosophies, and clinical settings. Long-term follow-up studies should contrast the long-term stability, periodontal health, andaesthetic outcomes of the AI-derived and human-derived treatment plans to establish whether ML models genuinely enhance patient outcomes or only reproduce existing variations. The addition of CBCT variables,such as the buccal bone thickness, the alveolar ridge width, and the distance of the root to the cortical plates, may also potentially improve the performance of the model, particularly for extraction decisions based on the assessment of the periodontal risk. Deep learning models that take raw lateral cephalograms or dental photographs, or 3D facial scans as input could potentially obviate manual feature extraction, and reveal novel radiographic phenotypes associatedwith best outcomes. In spite of these limitations, the present study demonstrates that ensemble ML could well mimic complex orthodontic extraction decisions with high accuracy while still being interpretable through feature importance analysis, which provides a strong foundation for thefuture development of practical toolsets for guiding clinical decision making in orthodontics.

## Conclusions

Ensemble ML models in particular Random Forest provides very good performance in modeling orthodontic extraction prediction with 93.5% accuracy and an AUC-ROC of 0.95 in this retrospective investigation. Mandibular crowding and lower incisor proclination (IMPA) are the strongest physical drivers of extraction therapy and account for 50% of the predictive weight, which is a statement of thebiomechanical boundaries of the mandible and periodontium. The application of ML as a CDSS may serve to standardize diagnosis, reduce human bias, and enhance agreement in treatment planning, especially in the "borderline" or low inter-clinician agreement diagnoses. To progress further, the integration of 3D CBCT imaging and longitudinal outcome data will be necessary to move beyond a human imitator to a biologically optimal decision support system.

## Data Availability

The datasets used and/or analyzed during the current study are available from the corresponding author.
